# In vitro analysis of the proliferative capacity and cytotoxic effects of ex vivo induced natural killer cells, cytokine-induced killer cells, and gamma-delta T cells

**DOI:** 10.1186/s12865-015-0124-x

**Published:** 2015-10-12

**Authors:** Chao Niu, Haofan Jin, Min Li, Jianting Xu, Dongsheng Xu, Jifan Hu, Hua He, Wei Li, Jiuwei Cui

**Affiliations:** Cancer Center, the First Hospital of Jilin University, 71 Xinmin Street, Changchun, 130021 China

**Keywords:** Natural killer cells, Cytokine-induced killer cells, Gamma-delta T cells, Cytotoxicity

## Abstract

**Background:**

Recent studies have focused on the significant cytotoxicity of natural killer (NK) cells, cytokine-induced killer (CIK) cells, and gamma-delta (γδ) T cells in tumor cells. Nevertheless, the therapeutic features of these cell types have not been compared in the literature. The aim of this study was to evaluate the feasibility of activation and expansion of NK, CIK, and γδ T cells from cancer patients in vitro, and to clarify the differences in their antitumor capacities.

**Methods:**

NK, CIK, and γδ T cells were induced from the peripheral blood mononuclear cells of 20 cancer patients by using specific cytokines. Expression of CD69, NKG2D, CD16, granzyme B, perforin, IFN-γ, and IL-2 was measured by flow cytometry. Cytokine production and cytotoxicity were analyzed by enzyme-linked immunosorbent assay and Calcein-AM methods.

**Results:**

NK cell proliferation was superior to that of CIK cells, but lower than that of γδ T cells. NK cells had a much stronger ability to secrete perforin, granzyme B, IFN-γ, and IL-2 than did CIK and γδ T cells, and imparted significantly higher overall cytotoxicity.

**Conclusions:**

Expanded NK cells from cancer patients are the most effective immune cells in the context of cytokine secretion and anti-tumor cytotoxicity in comparison to CIK and γδ T cells, making them an optimal candidate for adoptive cellular immunotherapy.

## Background

Cellular immunity plays an essential role in anti-tumor activity. Immunocyte activity is often compromised in tumor patients, due to the inhibitory tumor environment. Therefore, numerous preliminary studies have demonstrated the safety and efficacy of adoptive cellular immunotherapy (ACI)—consisting of ex vivo expansion and activation of patient immunocytes followed by reinfusion [1]. ACI has become a promising, innovative strategy for personalized cancer therapy [2]. Due to the complexity of immune escape—such as antigen loss, MHC class I down-regulation, and the expression of inhibitory molecules—specific immune cells such as cytotoxic T lymphocytes (CTL) have limited utility in cancer therapeutics [3]. Non-specific immune effector cells such as natural killer (NK) cells, cytokine-induced killer (CIK) cells, and gamma-delta (γδ) T cells have better potential, since they have no MHC restriction, contribute to front-line anti-tumor surveillance, and bridge the gap between innate and adaptive immunity [4, 5]. These cell types share many common mechanisms, including NKG2D and perforin-mediated cytotoxicity, and cytokine secretion [6–8]. However, each of them has unique features, and their therapeutic effects have been shown in ex vivo studies and clinical trials.

NK cells, which are large, granular CD3^−^CD56^+^ lymphocytes, can be rapidly activated to spontaneously attack certain abnormal cells in the body, particularly cancerous or virus-infected cells [9, 10]. Individuals who have low NK cell activity are at increased risk of developing cancer [11, 12]. Several clinical trials have confirmed the therapeutic benefit and safety of NK cell adoptive infusion [13, 14]. CIK cells are heterogeneous. The main effector cells are CD3^+^CD56^+^ T cells, which exhibit both the powerful anti-tumor effect of T cells and the non-MHC restriction of NK cells [15]. Recent clinical studies have indicated that CIK cells can significantly improve progression-free survival, overall survival, and effective clinical response in cancer patients [16]. In addition to NK and CIK cells, γδ T cells may display the same cytotoxicity as NK and CIK cells, and act as antigen-presenting cells.

For the past decade, ACI with NK, CIK, and γδ T cells has been a primary focus in cancer therapy, especially for hematological malignancies such as leukemia, lymphoma, and multiple myeloma [17–20]. However, there has been no direct comparison of these cell types. In this study, we evaluated the feasibility of in vitro expansion of each immune cell type and compared their antitumor activity against various forms of hematological cancers with the aim of providing in vitro evidence for their use in ACI cancer therapies.

## Results

### Cell quantification and expansion

**Fig. 1:**
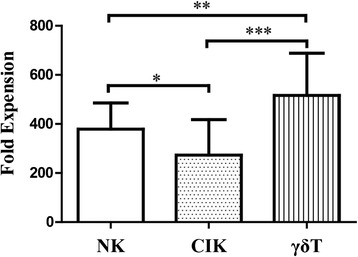
Fold expansion of NK, CIK, and γδ T cells. Fold expansion was calculated by dividing the absolute output number of expanded NK, CIK, and γδ T cells after 14 days of culture by the respective number on day 0. Results are expressed as mean ± SD, *n* = 20; * *p* < 0.05, ** 0.001 < *p* < 0.01, *** *p* < 0.001

NK, CIK, and γδ T cells were induced from the PBMCs of 20 cancer patients. After 14 days induction, immunocyte viability exceeded 85 % without contamination. All cells were able to propagate in vitro: NK cells to a median of 359-fold (250–669-fold, *n* = 20), CIK cells to 253-fold (120–621-fold, *n* = 20), and γδ T to 550-fold (159–879-fold, *n* = 20); γδ T cells had the strongest amplification ability (Fig. 1).

After induction and expansion, the percentage of expanded NK (*r* = 0.339, *p* = 0.001), CIK (*r* = 0.358, *p* = 0.003), and γδ T (*r* = 0.344, *p* = 0.004) cells showed a slight positive correlation with their percentage in the patients’ blood (Spearman’s test). These data suggest the percentage of immune cells in the patients’ blood likely has little effect on the ex vivo induction of these immune cell types, further demonstrating the stability and broad applicability of our methods.

### Immunophenotyping of expanded immune cells

**Fig. 2:**
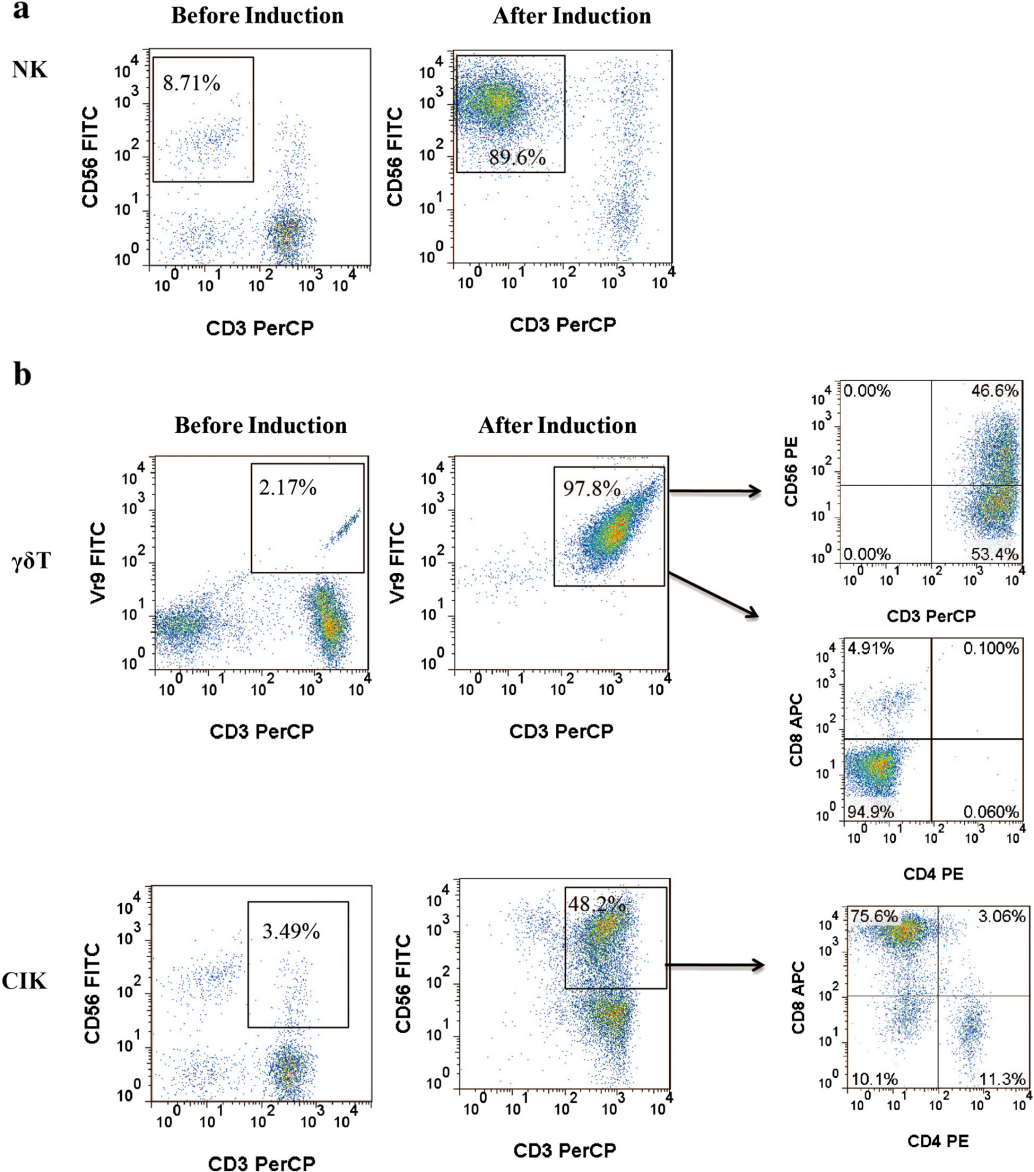
The percentage of NK, CIK, and γδ T cells before and after induction. **a** The percentage of NK cells (CD3^−^CD56^+^) before and after induction. **b** The percentage of γδ T (CD3^+^Vγ9^+^) and CIK (CD3^+^CD56^+^) cells before and after induction. γδ T and CIK cells were immunostained with CD4 and CD8. Representative results from a single patient are shown

The percentages of NK (CD56^+^CD3^−^), γδ T (Vγ9^+^CD3^+^), and CIK (CD56^+^CD3^+^) cells before and after induction were 16.1 (1.7–29.7) vs. 79.7 (62.1–98.4), 4.4 (0.9–10.2) vs. 65.9 (40.2–98.2) and 12.7 % (1.6–21.1 %) vs. 35.4 % (16.3–55.6 %), respectively. A portion of γδ T cells (10.2–45.9 %) was CD56-positive, and nearly no expression of CD4 or CD8 was observed in the population. However, the majority of CIK cells were CD8-positive (Fig. 2).

**Fig. 3:**
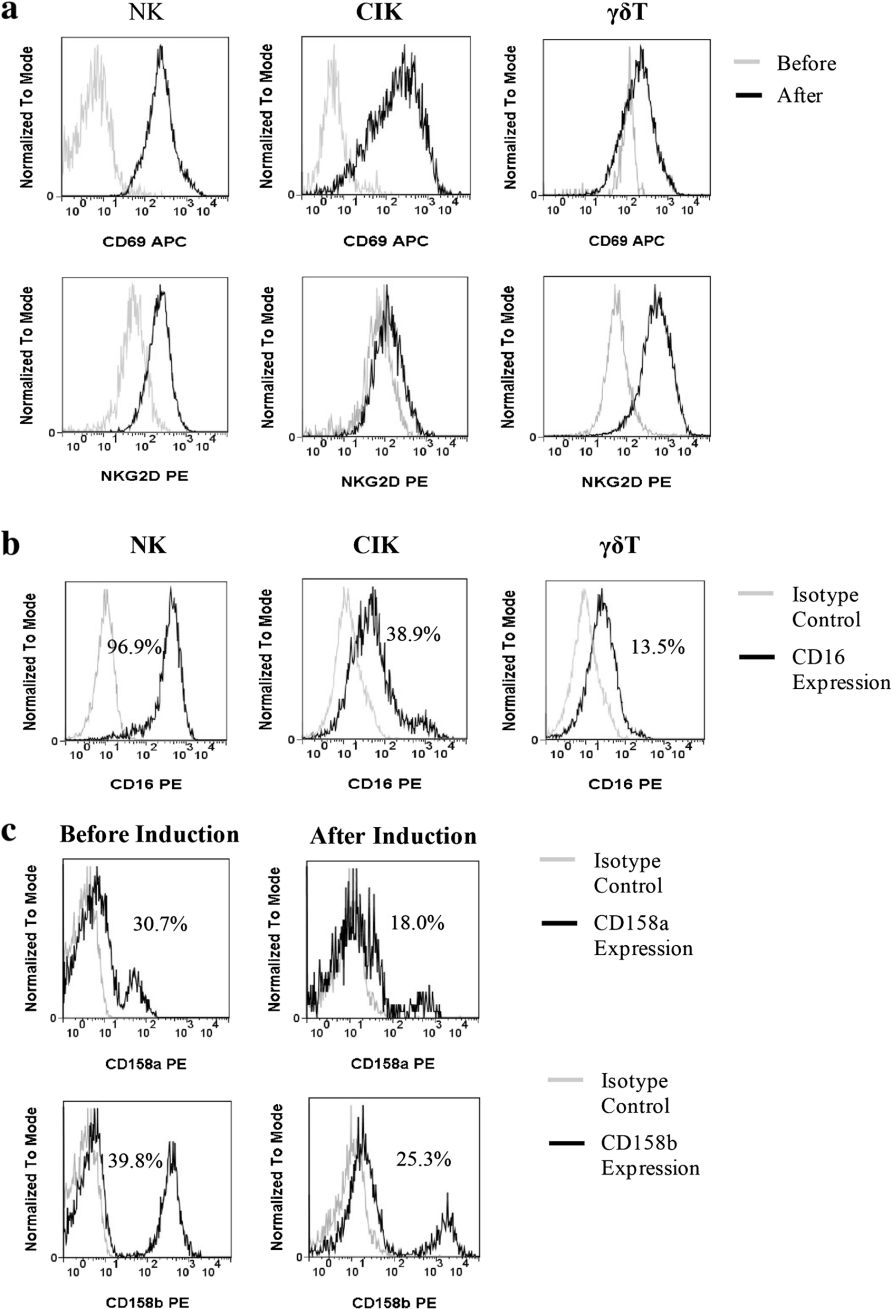
Expression of CD69, NKG2D, and KIR on induced immune cells. **a** The expression of CD69 and NKG2D were analyzed by flow cytometry (gray histogram). CD69 and NKG2D expression peaks are shifted to the right in expanded cells versus pre-induction cells (black histogram). **b** Expression of CD16; gray histograms depict isotype controls. **c** Expression of KIR (CD158a and CD158b) on NK cells before and after induction. Gray histograms depict isotype controls and the black histogram depicts the specific antibody. Data from one representative patient is shown

NK and CIK cells in patient blood rarely express CD69, but γδ T cells exhibited high expression of this marker. After induction and expansion, activated NK and CIK cells expressed much higher levels of CD69. However, there was only a slight increase in CD69 expression on γδ T cells before and after induction. After NKG2D induction, only γδ T cells showed a very strong increase (Fig. 3a). However, CD16 expression differed between immune cell types. NK cells had the highest CD16 expression at 78.7 % (50.2–98.2 %). In contrast, some of the CD3^+^CD56^+^ CIK cells (30.7, 6.9–59.6) and γδ T cells (11.7 %, 2.6–37.7 %) were also CD16-positive (Fig. 3b). After induction, inhibitory KIR CD158a and CD158b on NK cells were down-regulated. Representative results from one of these patients are shown in Fig. 3c.

**Fig. 4:**
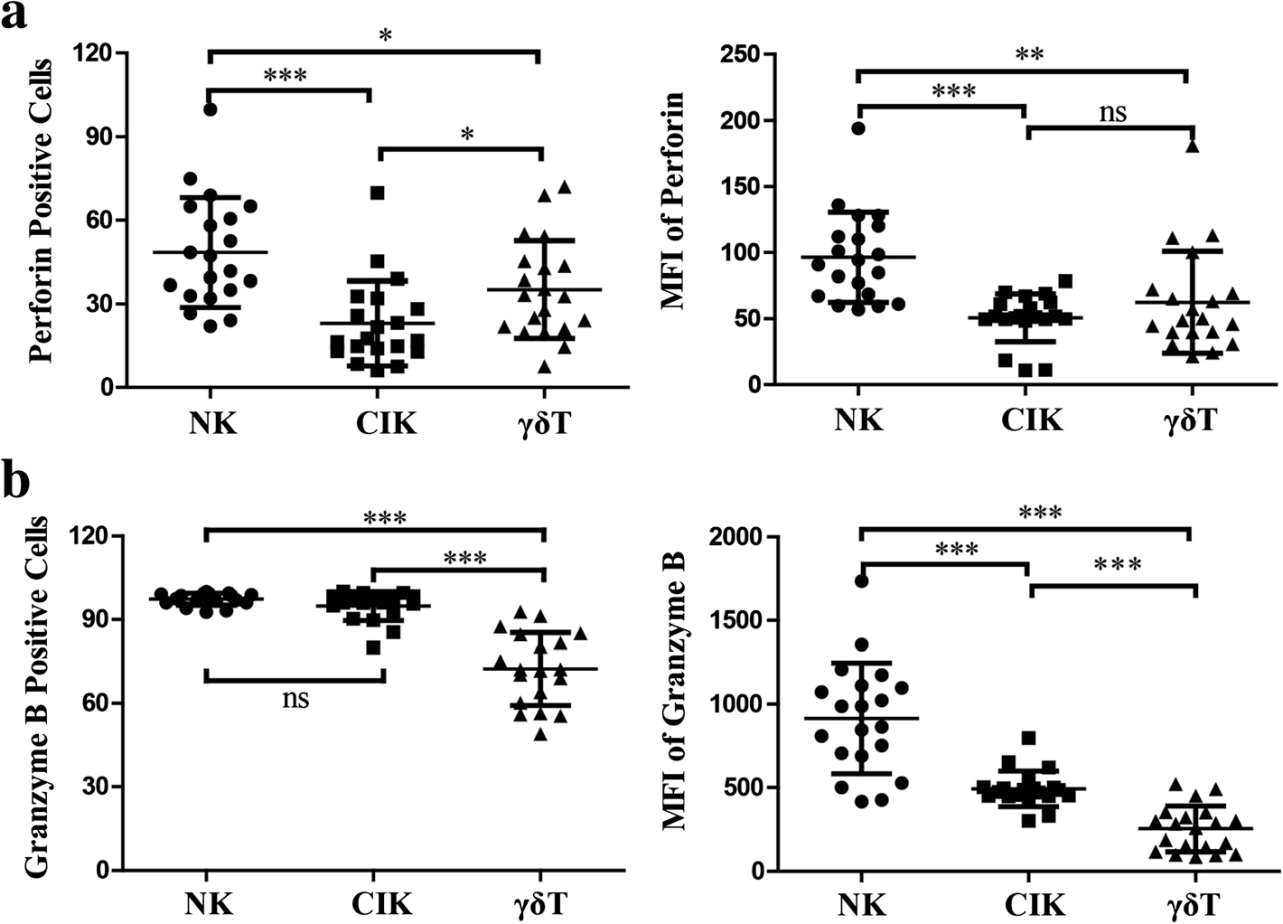
Perforin and granzyme B production in expanded NK, CIK, and γδ T cells. Three types of immune cells from twenty cancer patients were harvested after 14 days induction in vitro. NK and CIK cells were stained with mAbs to CD3 and CD56, and γδ T cells were stained with CD3 and Vγ9. After fixation and permeabilization, cells were stained for perforin and granzyme B using specific conjugated anti-human cytokine mAbs. **a** Perforin-positive cells and mean fluorescence intensity (MFI) of NK, CIK, and γδ T cells. **b** Granzyme B-positive cells and MFI of NK, CIK, and γδ T cells. Results are expressed as mean ± SD, *n* = 20. * *p* < 0.05, ** 0.001 < *p* < 0.01, *** *p* < 0.001, ns *p* > 0.05

All three induced immune cells expressed perforin. NK cells showed much higher perforin production than the other two expanded immune cell types. γδ T cells showed slightly higher perforin production than CIK cells (Fig. 4a). Almost all NK (97.3 ± 2.2) and CIK (94.8 % ± 5.2 %) cells and a majority of γδ T cells (72.3 % ± 13.1 %) were granzyme B-positive. However, NK cells yielded the strongest expression of granzyme B (Fig. 4b).

### Cytokine secretion of the expanded cells

**Fig. 5:**
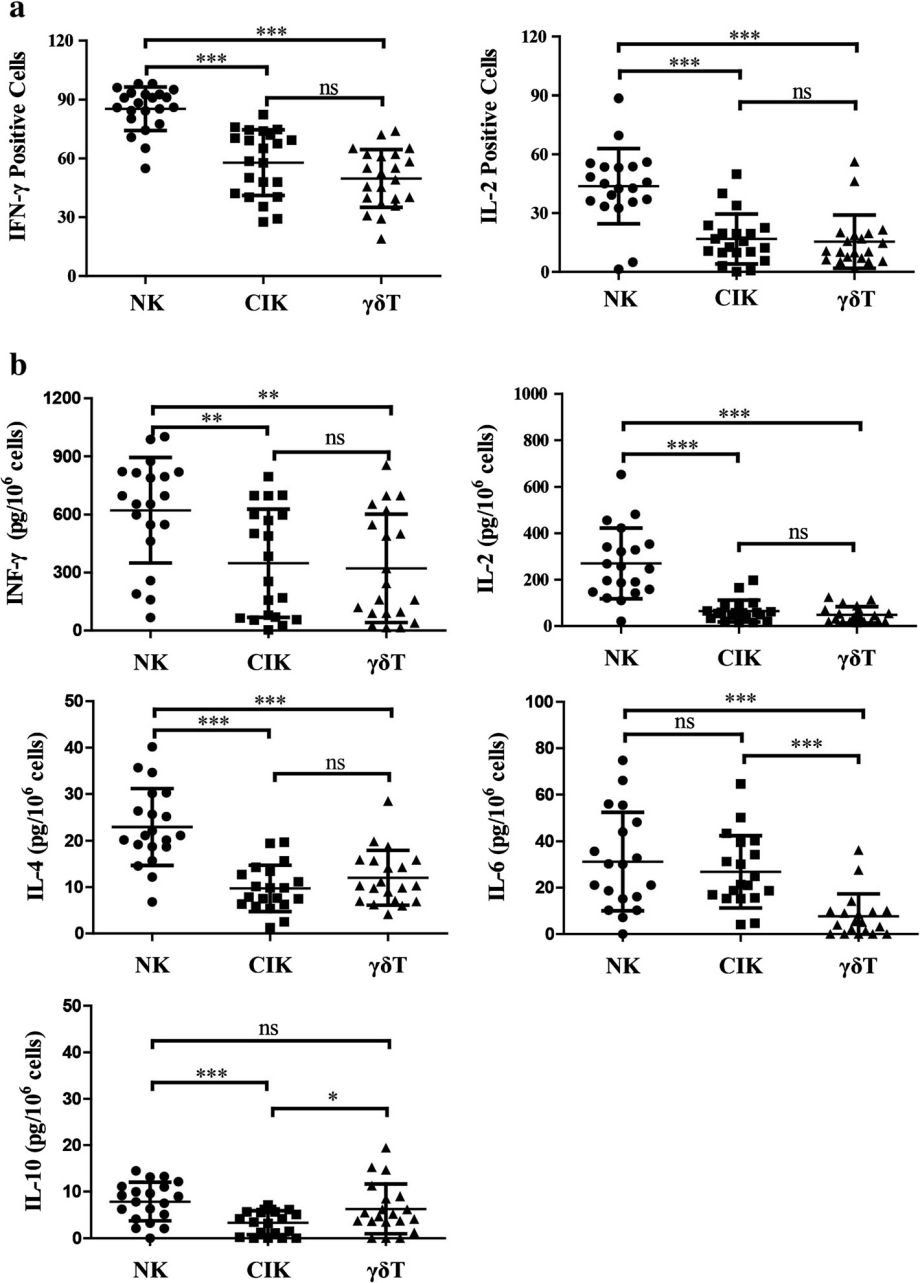
Cytokine production in NK, CIK, and γδ T cells. **a** Intracellular staining of IFN-γ and IL-2 in NK, CIK, and γδ T cells. Intracellular levels of IL-2 and IFN-γ were measured as described for perforin and granzyme B detection. **b** Extracellular cytokine production of NK, CIK, and γδ T cells. The culture supernatants were collected and analyzed by ELISA for IFN-γ, IL-2, IL-4, IL-6, and IL-10. Results are expressed as the mean ± SD, *n* = 20. * *p* < 0.05, ** 0.001 < *p* < 0.01, *** *p* < 0.001, ns *p* > 0.05

Nearly all of the expanded NK cells and half of the CIK and γδ T cells were IFN-γ-positive. About 40 % of the NK cells were IL-2-positive, while only a small portion of CIK and γδ T cells were positive for IL-2 (Fig. 5a). NK cells were much more efficient secretors of IFN-γ and IL-2 than were CIK and γδ T cells (Fig. 5b). Subsets of NK cells or CIK and γδ T cells secreted a very high level of IFN-γ, yet only a small amount of IL-2 was detected in the supernatant of the CIK and γδ T cells. No differences in INF-γ and IL-2 secretion were observed between CIK and γδ T cells. Very low levels of IL-4, IL-6, and IL-10 were detected in the supernatants of NK, CIK, and γδ T cells (Fig. 4b).

### Cytotoxicity of expanded immune cells against hematologic malignancies cell lines

**Fig. 6:**
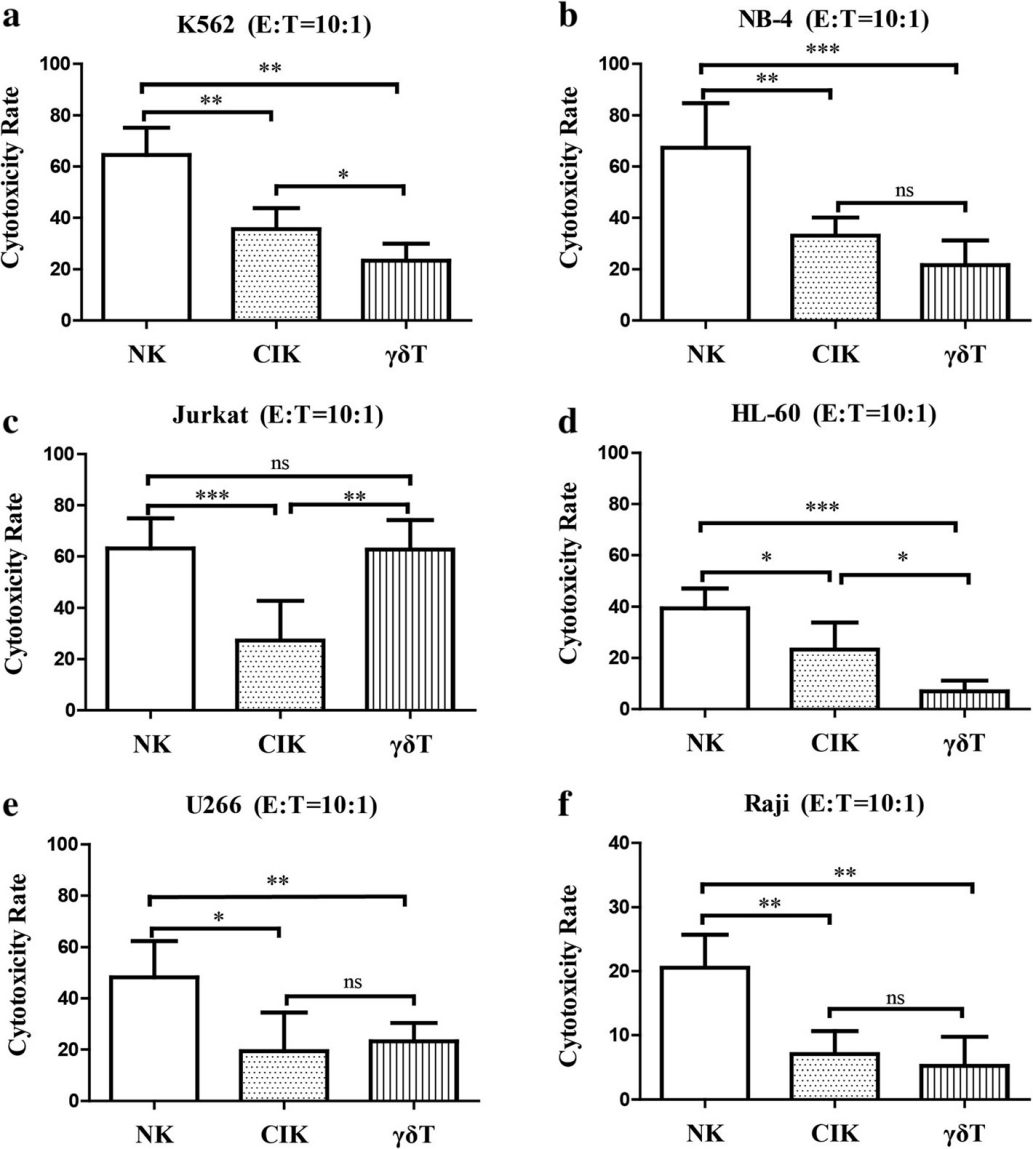
Anti-tumor cytotoxicity of induced immune cells in vitro. Cytolysis against hematological malignancy targets K562 (**a**), NB-4 (**b**), Jurkat (**c**), HL-60 (**d**), U266 (**e**), and Raji (**f**) cells were compared at 10:1 effector-target ratios (E:T). NK cells showed the most significant cytotoxicity. Results are expressed as the mean ± SD, *n* = 20. * *p* < 0.05, ** 0.001 < *p* < 0.01, *** *p* < 0.001, ns *p* > 0.05

**Fig. 7:**
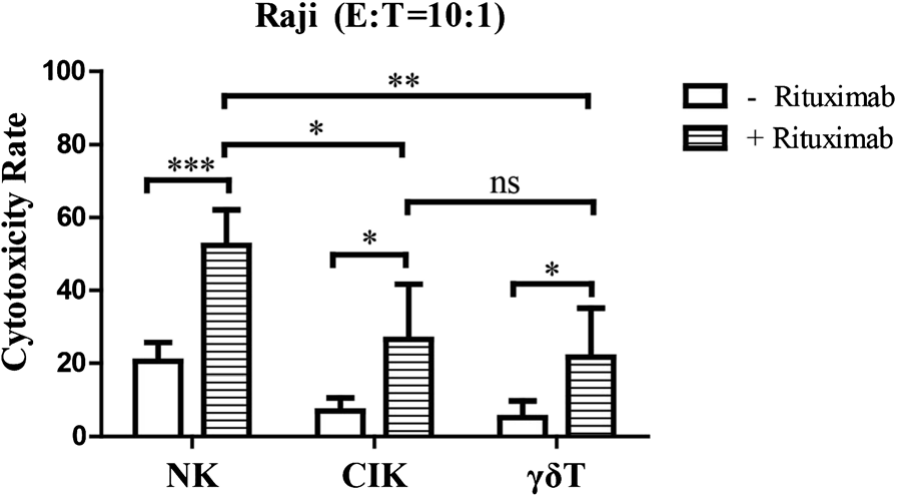
Antibody-dependent cellular cytotoxicity of induced NK, CIK, and γδ T cells. Raji cells were pre-coated with rituximab (Genentech) at 10 mg/mL for 30 min. The ADCC capacity at 10:1 effector-target ratios (E:T) were measured by the calcein-AM method. Effector cells were bulk samples of the cultured cells (i.e., no cell sorting was done). Results are expressed as mean ± SD, *n* = 20. * *p* < 0.05, ** 0.001 < *p* < 0.01, *** *p* < 0.001, ns *p* > 0.05

We compared the cytotoxicity of NK, CIK, and γδ T cells against hematologic tumors, as bulk expanded immune cells tested in the Calcein-AM assay showed substantial cytotoxic capacity. NK cells exhibited the most significant cytotoxicity in K562 (Fig. 6a), NB4 (Fig. 6b), HL-60 (Fig. 6d), and U266 (Fig. 6e) cells. Both NK and γδ T cells produced stronger cytotoxic effects against Jurkat cells than CIK cells (Fig. 6c). Although all three expanded cell types exhibited limited cytotoxicity against Raji cells compared to other target cells, NK cells exerted the strongest antitumor activity (Fig. 6f). Interestingly, the addition of rituximab significantly enhanced Raji lysis by all cell types; however, NK cells continued to show a greater synergistic cytotoxicity in Raji cells with rituximab in comparison to CIK and γδ T cells. Nevertheless, there were no notable differences in cytotoxicity against Raji cells between CIK and γδ T cells in the presence of rituximab (Fig. 7).

## Discussion

Although many studies have shown the importance of NK, CIK, and γδ T cells in the prevention of tumor relapse and metastasis in mice and humans [8, 21, 22], few studies have directly compared their cytotoxic effects to identify which would be the optimal candidate for clinical application. There are many issues to be considered prior to clinical application, including the number of introduced cells, the feasibility of in vitro expansion, and their respective anti-tumor activities.

Obtaining an adequate number of immune cells is a key challenge for ACI application in a clinical setting [23]. Studies indicate that sufficient numbers of CIK and γδ T cells can be obtained by current methods [3, 20]; however, methods for the expansion of NK cells in vitro have being investigated by using feeder cells, including irradiated allogenic PBMCs [24], K562 cells [25], and Epstein-Barr virus-transformed lymphoblastoid cell lines [19]. Although these NK cell infusions are well tolerated and partially effective, current methods for NK cell preparation involve complex separation protocols and often require ethical approval that render them expensive and sub-optimal or unfeasible for large-scale clinical studies [26]. To overcome these disadvantages, we examined whether the use of CD16 antibody-coated flasks could be used in place of feeder cells. Our studies demonstrate that a substantial number of NK cells could be generated, ranging from 250–669-fold expansions, after 14 days induction and culture. Importantly, this is comparable to the yields of feeder-cell methods [19]. In addition to NK cells, we amplified CIK and γδ T cells in vitro using conventionally optimized methods. Although several studies have shown that CIK cells easily proliferate in vitro [25], our investigation shows that the proliferative ability of NK cells in our culture setup was superior to that of CIK cells. We also found that the ratios of NK, CIK, and γδ T cells in the blood of cancer patients had no effect on their amplification efficiency. These results demonstrate that all three expanded immunocyte types have the potential for in vitro expansion to generate sufficient numbers for clinical applications.

The antitumor capacity of effector cells is another challenge in ACI. Our study assessed the phenotype, cytokine production, and cytotoxicity of the expanded cell types. Since NK, CIK, and γδ T cells have shown significant efficacy in the treatment of hematological malignancies in vivo and in vitro, we selected leukemia and multiple myeloma cell lines as target cells for our analyses.

There are two main NK cell subsets distinguished by the level of CD56 expression in human blood, namely CD56^dim^ and CD56 ^bright^ [27]. CD56^dim^ cells are fully matured and strongly express CD16, a low-affinity Fc immunoglobulin G receptor that allows immune cells to participate in ADCC [10]. In contrast, CD56^bright^ cells are more immature and are primarily considered to be cytokine producers with a limited role in cytolytic responses [28]. The NK cells expanded in this study had very high CD16 (Fig. 3b) and CD56 expression, and may represent a subset of NK cells undergoing differentiation. NK cells also express inhibitory and stimulatory receptors, such as killer immunoglobulin-like receptor (KIR), CD16, and NKG2D. The cytotoxic reactivity of NK cells results from the integration of signals from the stimulatory and inhibitory receptors as they engage ligands on the target cell surface. Our studies show that NK cells propagated in CD16 antibody-coated flasks exhibit increased expression of NKG2D and CD69, and decreased expression of inhibitory KIR. Intracellular and extracellular cytokine detection indicated that NK cells were more potent producers of IFN-γ than CIK and γδ T cells (Figs. 4a and 5e). IFN-γ enhances antigen presentation by dendritic cells and stimulates production of the CXC chemokines MIG (CXCL9) and IP-10 (CXCL-10), which inhibit tumor angiogenesis and recruit CXCR3-bearing effector cells to tumor sites [29, 30]. Therefore, NK cells producing high amounts of IFN-γ could contribute greatly to tumor regression during cancer immunotherapy.

Since induced immunocytes are usually used for ACI without purification, these cultured cells were directly harvested for cytotoxicity detection. In comparison to CIK and γδ T cells, the NK cells expanded in this study were significantly more cytotoxic toward many kinds of malignant cells lines including NK-resistant Raji cells (Fig. 6f). Raji cells normally elude NK cell recognition due to their expression of inhibitory KIR ligands (HLA-Cw3 and HLA-Cw4) and a lack of NKG2D ligands (MICA) [31, 32]. The increased cytotoxic effects observed with our cultured NK cells likely result from their down-regulation of inhibitory KIR–CD158a and CD158b that engage HLA-Cw4 and HLA-Cw3, respectively.

ADCC is another mechanism by which cells convey antitumor activity, which is dependent on immunocyte CD16 expression. NK cells showed the highest expression of CD16 and the strongest ADCC efficacy in the presence of rituximab (Fig. 7). This implies that large-scale NK cell expansion can promote the development of an effective combination therapy for cancer. While few differences were observed in ADCC function between CIK and γδ T cells, a significant difference in CD16 expression was detected between CD3^+^CD56^+^ CIK and γδ T cells. Interestingly, CIK cells displayed a stronger cytotoxic effect toward K562 and HL-60 cells than γδ T cells, in contrast to our observations in Jurkat cells.

## Conclusions

In this study, we established a cytokine-based expansion system for NK cells with the aim of developing them for clinical application. NK cells not only have a higher expansion capacity, but also much stronger antitumor activity in cytokine production, director cytotoxicity, and ADCC effect. Our analyses found that NK cells exhibit stronger cytotoxicity against lymphoma, leukemia, and myeloma cells than expanded CIK and γδ T cells. Therefore, our study provides ex vivo evidence for the direct comparison of NK, CIK, and γδ T cells. We also provide experimental evidence for their clinical application in hematological malignancies.

## Methods

### Cells and culture

Human leukemia (K562, HL-60, NB4, and Jurkat) cells, multiple myeloma (U266) cells, and lymphoma (Raji) cells were cultured in RPMI-1640 medium (Gibco, USA) supplemented with 10 % heat-inactivated fetal bovine serum (Gibco), 100 U/mL penicillin, and 100 mg/mL streptomycin (Invitrogen, USA) at 37 °C in a humidified 5 % CO_2_ incubator.

### Isolation of peripheral blood mononuclear cells

Heparinized peripheral blood samples were obtained from 20 volunteer cancer patients. Blood samples were centrifuged at 1800 × *g* for 10 min and plasma was transferred to new tubes. Peripheral blood mononuclear cells (PBMCs) were isolated by density gradient centrifugation using Ficoll (Nycomed Pharma AS, Norway) at 800 × *g* for 30 min.

### Expansion of NK, CIK, and γδ T cells

NK cells were expanded as described [33]. Briefly, PBMCs were resuspended in AIM-V (Invitrogen) medium with 5 % auto-plasma, 500 U/mL IL-2, 2 ng/mL IL-15 (both from Miltenyi Biotec, Germany), and 1 μg/mL OK432 (Shandong Luya Pharmaceutical Co., China) at a concentration of 1 × 10^6^ cells/mL. PBMCs were cultured in flasks coated with anti-CD16 (Beckman, USA) for 24 h at 39 °C in a humidified 5 % CO_2_ atmosphere. The cells were cultured in AIM-V medium supplemented with 5 % auto-plasma, 1000 U/mL IL-2, and 2 ng/mL IL-15 at 37 °C for the next 13 days.

To generate CIK cells, PBMCs were cultured in AIM-V medium with 5 % auto-plasma at 37 °C with 1000 U/mL IFN-γ (Miltenyi Biotec). After 24 h, 100 ng/mL mouse anti-human CD3 monoclonal antibody (Peprotech, USA), 1000 U/mL IL-2, and 1000 U/mL IL-1α (Miltenyi Biotec) were added. Fresh complete medium and IL-2 supplement (1000 U/mL) were added every three days.

To amplify γδ T cells, PBMCs were cultured in complete medium with 1 μM zoledronate (Zoledronic Acid, Jilin Province Xidian Pharmaceutical Sci-Tech Development Co., China) and 400 U/mL human IL-2. Fresh complete medium and IL-2 supplement (400 U/mL) were added every 2 or 3 days.

### Quantification

Cell expansion was expressed as “fold expansion,” which was calculated by dividing the absolute output number of NK, CIK, and γδ T cells after 14 days of culture by their number on day 0. Absolute output numbers of these three immune cells were calculated by multiplying the total number of viable cells by the percentages of these three immune cells as determined by flow cytometry. Total viable numbers of NK, CIK, and γδ T cells were determined by the CASY cell counter (BioSurplus, USA).

### Immunophenotyping

The cultures were collected, washed, incubated for 15 min with mouse mAbs against human CD3-PerCP, CD56-FITC, or PE, CD69-APC, CD16-PE (BD Biosciences, USA), and NKG2D-PE (BioLegend, USA). NK cells were incubated with CD158a-PE and CD158b-PE (BD Pharmingen, USA), CIK cells were incubated with CD4-PE and CD8-APC (BD Biosciences) and γδ T cells were incubated with Vγ9-FITC (BD Pharmingen), CD4-PE, and CD8-APC. Isotype-matched antibodies were used as controls. Perforin and granzyme B detection was performed according to the BD Cytofix/Cytoperm™ Kit manual (BD Biosciences). Briefly, NK, CIK, and γδ T cells were harvested and adjusted to 1 × 10^6^ cells/mL in RPMI-1640 medium containing 10 % fetal calf serum, and incubated 0.1 % GolgiStop (BD Biosciences) for 4 h. After pre-incubation with 10 % normal human serum, cells were stained with mAbs to identify NK (CD3^−^CD56^+^), CIK (CD3^+^CD56^+^), and γδ T cells (CD3^+^Vγ9^+^), followed by intracellular staining for perforin-PE and granzyme B-PE (BD Pharmingen), and the corresponding isotype antibodies to determine intracellular cytokine levels.

Flow cytometry data acquisition was performed on a BD FACS Calibur (BD Biosciences) with Cell Quest Pro software. Analysis was performed with FlowJo software (Tree Star, USA).

### Cytokine secretion analysis

NK, CIK, and γδ T cells were collected and suspended (1 × 10^6^ cells/mL) in AIM-V medium and incubated at 37 °C for 24 h in a humidified atmosphere of 5 % CO_2_. Supernatants were collected for detection of IFN-γ, IL-2, IL-4, IL-6, and IL-10. Cytokine secretion was quantified using commercially available enzyme-linked immunosorbent assay (ELISA) kits. Intracellular cytokine levels of IL-2 and IFN-γ were measured as described above for perforin and granzyme B.

### Cytotoxicity analysis

NK, CIK, and γδ T cells were used as the effectors and leukemia cells (K562, HL-60, NB-4, and Jurkat), lymphoma cells (Raji), and multiple myeloma cells (U266) were used as targets. Briefly, target cells were collected, washed once with PBS, and suspended in PBS at 1 × 10^6^ cells/mL. Calcein-AM was added to a final concentration of 1 μM. Cells were incubated in a humidified atmosphere of 5 % CO_2_ for 30 min and then washed twice with PBS. For antibody-dependent cellular cytotoxicity (ADCC) assays, Raji cells were pre-coated with rituximab (Roche, Switzerland) at 10 mg/mL for 30 min. Effector cells were bulk samples of the cultured cells, i.e., no cell sorting was done. Lysis of the target cells by effector cells was assessed by Calcein release assay at a 10:1 effector-target ratio (E:T). After 4 h incubation, calcein release in the supernatant was assessed on a BioTek Synergy HT Microplate Reader (BioTek Instruments, USA). The percentage Calcein release was calculated according to the formula: % specific release = [(experimental release – spontaneous release)/(maximum release – spontaneous release)] × 100 %.

### Statistical analysis

Flow cytometry data were collected before and after induction and analyzed for correlation using Spearman’s test. Other data were analyzed by Wilcoxon’s rank-sum test in SPSS13.0 software. Results were considered significant at p ≤ 0.05.

### Informed consent/patient enrollment

In this study, 20 tumor patients were enrolled. Informed consent in accordance with the Declaration of Helsinki was obtained from all patients, and approval was obtained from the Ethics Committee of the the First Hospital of Jilin University (protocol #2010–019). Inclusion criteria were no radiation or chemotherapy for at least one month before blood collection. Only patients with solid tumors were included. Patient characteristics are listed in the Table 1.

**Table 1:** Clinicla data of the patients in the study

Patients no.	Gender	Age	Type of tumor	Stages of disease
1	Female	36	Gastric Cancer	IB(T2N0M0)
2	Male	65	Gastric Cancer	IV(T4bN0M1)
3	Male	80	Gastric Cancer	IV (rcTxNxM1)
4	Female	71	Gastric Cancer	IIA (T3N0M0)
5	Female	39	Gastric Cancer	IIB (T1bN3aM0)
6	Male	66	Kidney Cancer	(T1aNxMx)
7	Male	62	Kidney Cancer	(T4NxM0)
8	Female	59	Breast Cancer	IV (pT1N1M1)
9	Female	49	Breast Cancer	I (pT1N0M0)
10	Female	49	Breast Cancer	I (pT1cN0M0)
11	Female	56	Breast Cancer	I (pT1N0M0)
12	Male	61	Liver Cancer	C (BCLC)
13	Male	71	Liver Cancer	IV (Intrahepatic cholangiocellular carcinoma)
14	Male	80	Liver Cancer	Early A (BCLC)
15	Female	63	Lung Cancer	IB (pT2aN0M0)
16	Male	50	Lung Cancer	IIIA (pT2aN2M0)
17	Male	43	Lung Cancer	IB (pT2N0M0)
18	Male	68	Lung Cancer	IIB (pT3N0M0)
19	Female	56	Lung Cancer	(pTisN0M0)
20	Female	69	Lung Cancer	Extended stage
